# A Survey of Lithuanian Pregnant Women’s Knowledge about Periodontal Disease, Its Prevalence and Possible Influence on Pregnancy Outcomes

**DOI:** 10.3390/medicina60091431

**Published:** 2024-09-01

**Authors:** Egle Ramanauskaite, Vita Maciulskiene, Nomeda Baseviciene, Rugile Anuzyte

**Affiliations:** Clinic of Dental and Oral Pathology, Lithuanian University of Health Sciences, 44307 Kaunas, Lithuania; egle.ramanauskaite@gmail.com (E.R.); nomeda.baseviciene@lsmu.lt (N.B.); anuzyteee@gmail.com (R.A.)

**Keywords:** pregnancy, periodontal disease, gingivitis, periodontitis, periodontal health, prevalence, negative pregnancy outcomes

## Abstract

*Background*: This study aimed to subjectively assess the changes in the oral health status of pregnant women, to find out their attitudes and knowledge about possible changes in the oral cavity that occurred during pregnancy, and their influence on the outcomes of pregnancy. *Methods*: 112 pregnant women who visited the Republican Siauliai Hospital during their pregnancy participated in the study. An anonymous self-administered questionnaire was used to conduct the research, surveying their demographic characteristics, oral health changes and habits during pregnancy, and awareness of possible negative pregnancy outcomes. *Results*: The study involved 112 pregnant women, with 35 in the first trimester, 28 in the second, and 48 in the third trimester. The findings revealed that pregnant women do not take sufficient care of their oral health: more than half of the respondents did not visit an oral care specialist during pregnancy; 22.3% of women reported brushing their teeth only once a day or less; and 35.7% did not clean interdental surfaces at all. Statistically, significantly more urban women believed there is a relationship between maternal oral health and adverse pregnancy outcomes than women living in rural areas (*p* = 0.013). While significant oral health changes were not observed in the first trimester, more women in the second and third trimesters reported issues such as bleeding gums, swelling, plaque accumulation, tartar, and caries (*p* < 0.001). *Conclusions*: The attitude of women towards oral health during pregnancy and their understanding of the possible risks for unfavorable pregnancy outcomes are insufficient. Notably, oral health issues become more prominent in the second and third trimesters, necessitating appropriate oral care to reduce the incidence of oral and dental diseases during pregnancy. This underscores the importance of educational and preventive public health policies focused on oral care for pregnant women, aimed at increasing awareness and promoting practices that safeguard both maternal and fetal health.

## 1. Introduction

Periodontal diseases, such as gingivitis and periodontitis, are one of the most common diseases in the world among adults, and the severity of the diseases progresses with age [1]. According to research, more than every second person has some signs of periodontal disease [2,3]. It is often observed that the health of a woman’s mouth begins to deteriorate already when expecting a newborn [4]. Pregnancy is associated with gingivitis, gingival bleeding, swelling, and sometimes mild pain, as well as with periodontitis, characterized by increased gingival pocket depth, radiographic alveolar bone loss, and clinical attachment loss [5,6,7].

It is well established that pregnancy causes numerous hormonal, metabolic, and immunological changes in women’s bodies that impact the composition of the oral microbiome [8]. In the oral cavity, there is normally a balance between microbial invasion and the ability of the immune system to overcome it, but when this balance is disturbed by local or systemic factors, inflammation of the surrounding tissues begins. Periodontal disease is an infection of bacterial origin that progresses, especially with the accumulation of plaque on the teeth, and the process is even faster with hormonal changes during pregnancy. Many tissues undergo some changes as the placenta secretes higher levels of estrogen and progesterone. During pregnancy, estrogen and progesterone promote gingival oedema and dilation of blood vessels. Periodontal disease is an inflammatory response to dental plaque that can lead to tooth loss if left untreated [9,10]. The first signs of gingivitis often begin in the second month of pregnancy, and the peak is reached in the eighth month, and usually after the birth of the child, the health of the gums stabilizes [10,11].

The oral cavity contains the second most complex microbial population in the human body, which is a natural niche for more than 600 different species of streptococci, lactobacilli, staphylococci and other microorganisms [12]. Oral microorganisms have been found to cause several infectious oral diseases such as tooth decay, gingivitis, root canal infection, alveolitis, and tonsillitis [12]. There is a growing body of evidence that reveals that oral bacteria also cause systemic disorders [8]: cardiovascular disease [13,14], stroke [15,16], premature birth [6,17], diabetes [18], and even cases of pneumonia [19]. When examining the oral microbiome, studies show that the total number of oral microbes during pregnancy is higher than in a non-pregnant woman. It has been observed that *Porphyromonas gingivalis*, *Aggregatibacter actinomycetemcomitans*, *Streptococci*, *Staphylococci*, and *Candida* species are found much more often in pregnant women, especially in the first and second trimesters [20]. The dynamic change in the composition of the oral microbiome during pregnancy is multifactorial and occurs in part due to the complex changes that control a woman’s body during pregnancy [21].

We cannot deny the fact that women are not sufficiently informed about oral care during pregnancy and the risks associated with it. Educational and preventive guidelines are essential for safeguarding the health of pregnant women and their babies, reducing the risk of complications, and promoting long-term wellness. Pregnant mothers-to-be face an increased appetite due to a growing baby that requires a more varied and abundant diet [22]. Only half of pregnant women consult a dentist for a preventive examination. Interestingly, the reasons for not visiting a dentist are the absence of toothache, a lack of time, or even a lack of information about dental treatment options during pregnancy [5]. Based on research, systemic periodontal diseases can influence negative pregnancy outcomes, such as miscarriage, preeclampsia [23], premature birth [6], and low birth weight [24]. Therefore, there are recommendations for pregnant women and examinations related to the evaluation of oral health status in order to avoid possible consequences [5].

In Lithuania, the implementation of mandatory and/or recommended dental visits for pregnant women is outlined in national health regulations and guidelines. According to the Lithuanian Ministry of Health regulations, pregnant women are encouraged to seek dental care as part of their prenatal care routine. The guidelines emphasize the importance of oral health during pregnancy and recommend at least one dental check-up during pregnancy [25]. Data from the National Health Survey indicates that a significant portion of pregnant women in Lithuania utilize these services; however, the uptake is not universal. For instance, a recent survey reported that approximately 60% of pregnant women visited a dentist at least once during their pregnancy, but this figure suggests that a notable number of women may still lack regular dental care during this critical period. This underscores the need for enhanced public health initiatives to ensure that all pregnant women receive the recommended dental care to mitigate the risks associated with poor oral health [26].

The aim of this study is to evaluate changes in the oral health status of pregnant women, assess their attitudes and knowledge regarding oral health during pregnancy, and examine the potential impact of these factors on pregnancy outcomes.

## 2. Methods

### 2.1. Sample and Data Collection

A total of 112 pregnant women agreed to participate in the self-administered questionnaire study. The study was conducted at the Republic Hospital of Siauliai—the children’s and women’s clinic. To conduct the research, a permit was given by the Lithuanian University of Health Sciences Bioethics Committee on 30 November 2022 (BEC-OF-24). The investigation started on 1 December 2022and finished on 1 February 2022

The object of the study was pregnant patients of all trimesters. A questionnaire prepared in advance by the authors was used to conduct the study. In the preamble of the study, the patients were informed about the anonymous questionnaire, the purpose of the study was explained, and the respondents signed the informed consent form. Confidentiality of the subjects’ personal information was ensured throughout the study.

### 2.2. Study Sample Calculation

The study sample size was calculated using the Paniotto formula. It was determined that during the study period (1 December 2022–1 February 2023), an average of about 120 women gave birth per month at the Republican Siauliai Hospital. After calculations, with a 0.05 margin of error, the required sample size for the study was determined to be 92 patients.

### 2.3. Inclusion and Exclusion Criteria

Inclusion Criteria:

- Voluntary participation in the study;
- Pregnant women;
- Healthy individuals;
- Adults;
- Able to respond to written questions.

Exclusion Criteria:

- Women who have given birth;
- Minors;
- Unable to respond to written questions;
- Unwilling to cooperate.

### 2.4. Questionnaire

The questionnaire was anonymous and consisted of 37 questions with provided options; the names, addresses, or other personal information of the patients were not included in the study. The results of the study were published only in summary form. The questionnaire was divided into 3 parts. The questions of the first part were composed of social and demographic indicators (age, education, place of residence, number of pregnancies, trimester). The second part consisted of questions about the frequency of visits to an oral care specialist, the reasons why pregnant women did or did not visit an oral health specialist, and the importance of dental procedures during pregnancy. In the third part, questions related to changes in sensations in the oral cavity during pregnancy and the relationship between maternal oral health and negative pregnancy outcomes were presented.

### 2.5. Statistical Analysis

Summaries of the obtained data and charts were made using Microsoft Excel 2021 and IBM SPSS statistics (Statistical Package for the Social Science for Windows; Chicago, IL, USA) package 29.0. The probability level for significance was set at *p* < 0.05.

Using descriptive statistical methods, the frequencies and relative frequencies (expressed as percentages) of qualitative variables were calculated. Quantitative variables were described by determining the arithmetic mean, standard deviation, medians, 25–75 percentiles, and the minimum and maximum values. The interdependence of the qualitative variables was assessed using the Pearson Chi-Square (χ^2^) test.

## 3. Results

The sample size was 112 women with an age range of between 19 and 39 years (median 28.0 [24.3–31.0]) attending the Republican Hospital of Siauliai. The demographic and pregnancy characteristics are presented in Table 1. Mainly the pregnant women had completed higher education (28.6%). More than half (50.9%) lived in the city. Women in their 3rd trimester participated most actively in the survey (42.9%). For the majority of the women (42.9%), this was their first pregnancy.

The women’s perception of the importance of oral health and treatment options during pregnancy was limited (Table 2). Approximately 48% of the respondents stated that during pregnancy it is safe to see an oral health specialist, while 22% claimed that it is not safe, and 30% did not know. Only 25.9% of the women answered correctly that dental treatments are safest in the 2nd pregnancy trimester. During pregnancy, 41.1% of the women claimed that they went to see a dental hygienist, whilst 54.5% had seen a dentist. The women who visited the dentist were mostly treated for caries (39.3%), had prophylactic dental examinations (33%), dental hygiene (22.3%) or surgical treatments (11.6%). The reasons for not attending a dental check-up with an oral health specialist were the absence of information, which is essential for pregnant women (61.5%), that they had visited before pregnancy (44.2%), an absence of dental symptoms (40.4%), money shortage (19.2%), and the stress, fear, and pain they experienced (19.2%).

Oral health changes can vary during each trimester of pregnancy (Table 3). In the first pregnancy trimester, more than half of the women (52.8%) stated that they did not notice any changes in their oral health, or they had minor tooth sensitivity and bleeding gums. Statistically significant differences in oral status were observed during the second trimester. Some 57.1% claimed that they have tooth/gum sensitivity, 89.3% stated that they had observed bleeding gums, and 17 of the pregnant women had noticed gingival oedema and redness. Also, 57.1% of the pregnant women in their second trimester had observed increased dental plaque, calculus, and dental caries. Similar changes in the oral cavity are observed during the third trimester of pregnancy as in the second trimester in pregnant women. It is important to note that every woman’s experience during pregnancy can be different. While these oral health changes are common, not all women will experience them.

Oral health has been found to be associated with negative pregnancy outcomes. Poor oral health, particularly gum disease (periodontal disease), has been linked to an increased risk of certain adverse pregnancy outcomes. The results in Table 4 show that there is some uncertainty and lack of consensus among the respondents regarding the impact of hormones on periodontal disease. Only 33.0% of the respondents answered “Yes” when asked if hormones have an effect on periodontal disease. Many respondents (53.6%) recognized the importance of curing oral diseases before pregnancy, indicating a general understanding of the significance of oral health in relation to pregnancy. Some 25.0% of the respondents believed that there is no such relationship between maternal oral health and adverse pregnancy outcomes, and 50.0% of the respondents were uncertain or did not know. These results suggest a lack of agreement among the respondents regarding the relationship between maternal oral health and adverse pregnancy outcomes. While some respondents recognized specific complications, a significant portion either believed that poor oral health does not cause complications (35.7%) or were uncertain about the relationship (30.4%). Table 5 shows that statistically, significantly more woman that lived in a rural area believed that there is no relationship between oral health and negative pregnancy outcomes, compared to woman living in the city (14.0%) (*p* = 0.013). Additionally, women aged 31 years and older (45.2%) expressed a higher likelihood of perceiving a possible connection between oral health and pregnancy complications (*p* = 0.026).

Oral hygiene behavior during pregnancy is presented in Table 6. These results highlight that a majority of the respondents (76.8) adhere to a recommended toothbrushing frequency, while a notable portion (22.3) may benefit from improving their toothbrushing habits. Some of respondents practice proper interdental care, but a significant portion (35.7) neglects this aspect of oral hygiene, highlighting the need for education and awareness. Dental professionals, both dental hygienists and dentists, play a crucial role in educating pregnant women about proper oral hygiene practices during pregnancy. However, there is room for improvement in promoting oral health during pregnancy, and 30.4% were not instructed how to maintain proper oral hygiene. There was a lack of consensus among the respondents regarding the frequency of dental visits during pregnancy, with a significant proportion suggesting that visits should be based on symptoms (50.0%) rather than regular check-ups. A small percentage of the respondents also expressed concerns about the safety of dental visits during pregnancy.

## 4. Discussion

Monitoring a woman’s oral health during pregnancy is very important both for the mother’s well-being and for the growing fetus. The data from the conducted study showed that 41.1% of the pregnant women visited a dental hygienist, while 54.4% visited a dentist during pregnancy. Meanwhile, according to the data from a study conducted in Kaunas city in 2017, 68.3% of the participating respondents visited a dentist during pregnancy [27]. Analyzing the results of a study conducted in France, we can see that 47% of pregnant women visited a dentist, and 25% of them received dental treatment [5]. Interestingly, French women did not visit oral care specialists because they did not experience any changes or symptoms (42.7%), lacked time (25%), or did not have information about the need for careful oral health during pregnancy (14%) [5]. The data from our study shows that women mostly visited oral care specialists for dental fillings (39.3%), preventive check-ups (33%), and professional oral hygiene (22.3%). Women did not visit dentists because nobody advised them to do so (61.5%), they visited before getting pregnant (44.2%), or they had no complaints (40.4%).

In scientific studies, hypotheses are raised regarding the influence of oral cavity diseases on pregnancy and its course, suggesting that poor oral health can contribute to low birth weight, premature birth, preeclampsia, miscarriage, or fetal developmental disorders [6,23,24]. The data from the conducted study indicate that 25% of pregnant women believe there is a relationship between maternal oral health and adverse pregnancy outcomes, 25% claim there is no connection, while the majority of women (50%) are unaware of any associations. Some 8.9% of women believed that poor oral health could pose a risk of miscarriage and fetal developmental disorders, while 8% believed it could result in lower birth weight or premature birth. The data from a study conducted in 2015 show that 18.2% of the pregnant women believed in a correlation between oral health and adverse pregnancy outcomes, 22.2% believed there was no connection, and 58.6% of the women stated that they had never even heard of such a possible association [28]. A study conducted in India in 2013 revealed that 87.2% of the women were unaware of the importance of oral health during pregnancy and its potential negative impact on pregnancy outcomes [29]. It can be concluded that pregnant women lack sufficient information about the possible effects of oral cavity health on adverse pregnancy outcomes.

Different changes in the oral cavity are observed during different trimesters of pregnancy, indicating various conditions such as gingivitis, gingival hyperplasia, pregnancy epulis, and saliva changes [30,31,32]. The results of the conducted study revealed that in the first trimester, 52.8% of women did not experience any changes in their oral health, 19.4% experienced bleeding gums, and 11.1% developed plaque and tartar. In the second and third trimesters, significantly more women complained of bleeding gums (89.3% and 79.2%, respectively), swollen gums (60.7% and 39.6%), dental decay (57.1% and 37.5%), and plaque and tartar (57.1% and 41.7%). According to the data from a study conducted in 2019, the majority of women (37.75%) reported toothache, 28.57% mentioned bleeding gums, 17.34% indicated tooth sensitivity, and 7.65% reported swollen gums. The study evaluated the OHI (Oral Hygiene Index), GI (Gingival Index), and PI (Periodontal Index) scores in different trimesters. A statistically significant difference was observed in the OHI index between trimesters, with scores of 1.9896 in the first trimester, 2.1685 in the second trimester, and 2.073 in the third trimester (*p* < 0.01). Statistically significant differences were also found in the GI scores: 0.88 in the first trimester, 1.4098 in the second trimester, and 1.837 in the third trimester (*p* < 0.01). Likewise, the PI score showed significant variations: 0.674 in the first trimester, 1.46605 in the second trimester, and 1.654 in the third trimester [33]. Comparing the data from different studies, we can observe a trend where no significant oral cavity changes are noticed during the first trimester or only mild gum inflammation is observed. However, an increase in plaque, bleeding gums, tooth sensitivity, and tooth decay is seen in the second and third trimesters.

Maintaining proper individual oral hygiene during pregnancy is crucial to prevent the development of oral cavity diseases. The data from our study show that 22.3% of the pregnant women reported brushing their teeth once a day, while 76.8% stated they brushed their teeth 2–3 times a day. Interdental care was as follows: 45.5% used dental floss, 8.9% used interdental brushes, 17.0% used oral irrigators, and 35.7% did not clean between their teeth. Comparing this to a study conducted in the Netherlands, out of 187 women, 72.2% brushed their teeth twice a day. Interdental cleaning was practiced by 62.0% of women, primarily using dental floss (31.0%) or wooden interdental sticks (32.6%). However, only 23.3% cleaned between their teeth daily, with a majority engaging in interdental care only a few times a week (29.3%), once a week (13.8%), or even less frequently than once a week (17.2%) [34]. A study conducted in France indicates that 74% of women brushed their teeth twice a day, while 24% brushed more than twice a day [5]. Analyzing the obtained results, it can be observed that brushing teeth twice a day is an acceptable practice for most women, but interdental cleaning is insufficient.

These findings underscore the importance of implementing educational and preventive guidelines for pregnant women. Such guidelines are essential for increasing awareness about the critical role of oral health during pregnancy, encouraging regular dental visits, and promoting effective oral hygiene practices. By providing pregnant women with the necessary knowledge and tools, healthcare providers can help prevent oral health issues and reduce the risk of adverse pregnancy outcomes, ultimately ensuring better health for both mothers and their babies.

The study has several limitations that may impact its findings. The small sample size of 112 pregnant women from a single hospital limits the generalizability of the results to a broader population. The reliance on self-reported data introduces the possibility of response bias, and the lack of objective oral health assessments prevents corroboration of these reports with clinical data. The cross-sectional design restricts the ability to assess changes in oral health over time or establish causality between oral health and pregnancy outcomes. Potential recall bias may affect the accuracy of the participants’ reports, especially in later pregnancy stages. The study also did not explore the impact of sociocultural factors, which could influence oral health behaviors and care-seeking. Additionally, focusing on subjective perceptions without objective follow-up limits the understanding of the clinical significance of reported oral health changes. Lastly, the study’s exclusion of non-hospitalized pregnant women, by only including those who visited the Republican Siauliai Hospital, could introduce selection bias, as the sample may not represent the full spectrum of pregnant women, particularly those with different levels of healthcare access.

## 5. Conclusions

The study underscores the crucial need for public policies that guide pregnant women on oral hygiene care. The findings reveal a significant gap in knowledge about oral health and inadequate oral hygiene practices among pregnant women. Many pregnant women do not regularly visit dental professionals, resulting in a lack of awareness regarding the potential risks poor oral health poses to pregnancy outcomes. Although most women in the first trimester did not observe oral health changes, those in the second and third trimesters reported issues such as bleeding gums, swelling, plaque and tartar buildup, and dental decay. This highlights the necessity for educational and preventive guidelines to improve awareness, promote routine dental visits, and encourage proper oral hygiene practices. Implementing such guidelines is essential to reduce the risk of adverse pregnancy outcomes and enhance both maternal and fetal health.

## Figures and Tables

**Table 1:** Demographics.

Clinical Parameter	Total (n = 112) n (%)
**Educational level**	
Primary school	7 (6.3)
Secondary school	24 (21.4)
Vocational	27 (24.1)
Collegiate	22 (19.6)
University	32 (28.6)
Residence	
City	57 (50.9)
District	27 (24.1)
Village	28 (25.0)
**Trimester of pregnancy**	
1st	36 (31.3)
2nd	28 (25.0)
3rd	48 (42.9)
**Number of pregnancies**	
1st	48 (42.9)
2nd	41 (36.6)
3rd	21 (18.8)
4th	1 (0.9)
Other	1 (0.9)

**Table 2:** Awareness about dental treatments and their frequency during pregnancy.

Questions	Total (n = 112) n (%)
**Is it safe to see an oral care specialist during pregnancy?**	
Yes	54 (48.2)
No	25 (22.3)
Did not know	33 (29.5)
**Which trimester of pregnancy is the safest to have dental treatment?**	
1st	25 (22.3)
2nd	29 (25.9)
3rd	19 (17.0)
Teeth cannot be treated during pregnancy	16 (14.3)
Did not know	23 (20.5)
**Did you visit an oral hygienist during pregnancy?**	
Yes	46 (41.1)
No	66 (58.9)
**Did you visit a dentist during pregnancy?**	
Yes	61 (54.5)
No	51 (45.5)
**What dental procedures were performed during pregnancy?**	
Prophylactic dental examination	37 (33.0)
Periodontal treatment	0 (0.0)
Orthodontic treatment	4 (3.6)
Endodontic treatment	5 (4.5)
Dental bleaching	2 (1.8)
Surgical treatment	13 (11.6)
Dental hygiene	25 (22.3)
Tooth fillings and restorations	44 (39.3)
Other	2 (1.8)
**For what reasons did you not visit an oral health specialist during pregnancy?**	
No one advised me to	32 (61.5)
I visited before I got pregnant	23 (44.2)
No dental symptoms	21 (40.4)
Queues at doctors	2 (3.8)
Money shortage	10 (19.2)
It was not possible to get there	2 (3.8)
Worried about side effects	6 (11.5)
Stress, fear, pain	10 (19.2)
Nausea	1 (1.9)
No time	1 (1.9)

**Table 3:** Analysis of stated oral changes during pregnancy associated with pregnancy trimesters.

Question	1st Trimester (n = 36) n (%)	2nd Trimester (n = 28) n (%)	3rd Trimester (n = 48) n (%)	Pearson Chi-Square Exact Test, *p*-Value
**During Pregnancy Have You Noticed Any Changes in Your Oral Health?**				
Nothing	19 (52.8)	1 (3.6)	1 (2.1)	χ^2^ = 40.348, *p* < **0.001**
Tooth/gums sensitivity	12 (33.3)	16 (57.1)	21 (43.8)	χ^2^ = 3.628, *p* = 0.163
Bleeding gums	7 (19.4)	25 (89.3)	38 (79.2)	χ^2^ = 42.734, *p* < **0.001**
Gingival oadema, redness	0 (0.0)	17 (60.7)	19 (39.6)	χ^2^ = 28.750, *p* < **0.001**
Unpleasant smell	1 (2.8)	1 (3.6)	12 (25.0)	χ^2^ = 12.009, *p* = **0.002**
Gingival recession	0 (0.0)	0 (0.0)	2 (4.2)	χ^2^ = 2.715, *p* = 0.257
Reacts to thermal stimuli	0 (0.0)	2 (7.1)	2 (4.2)	χ^2^ = 2.420, *p* = 0.298
Erosions	0 (0.0)	0 (0.0)	1 (2.1)	χ^2^ = 1.345, *p* = 0.510
Caries	2 (5.6)	16 (57.1)	18 (37.5)	χ^2^ = 20.322, *p* < **0.001**
Dental plaque and calculus	4 (11.1)	16 (57.1)	20 (41.7)	χ^2^ = 15.832, *p* < **0.001**
Sensitive during tooth brushing	0 (0.0)	2 (7.1)	10 (20.8)	χ^2^ = 9.831, *p* = **0.007**

**Table 4:** Women’s knowledge about oral health during pregnancy and its influence on negative pregnancy outcomes.

Questions	Total (n = 112) n (%)
**Do hormones have an effect on periodontal disease?**	
Yes	37 (33.0)
No	41 (36.6)
Did not know	34 (30.4)
**Is it important to cure oral diseases before pregnancy?**	
Yes	60 (53.6)
No	17 (15.2)
Did not know	35 (31.3)
**Is there a relationship between maternal oral health and adverse pregnancy outcomes?**	
Yes	28 (25.0)
No	28 (25.0)
Did not know	56 (50.0)
**What pregnancy complications can be caused by poor oral health?**	
Low birth weight	9 (8.0)
Premature birth	9 (8.0)
Preeclampsia	5 (4.5)
Greater risk of child caries	8 (7.1)
Disorders of fetal development	10 (8.9)
Pneumonia	1 (0.9)
Miscarriage	10 (8.9)
Does not cause any complications	40 (35.7)
Did not know	34 (30.4)

**Table 5:** Knowledge of pregnant women about the association between oral health and adverse pregnancy outcomes according to their place of residence and age.

Question	City (n = 57) n (%)	District (n = 27) n (%)	Village (n = 28) n (%)	Pearson Chi-Square Exact Test, *p*-Value
**Is There a Relationship between Maternal Oral Health and Adverse Pregnancy Outcomes?**				
Yes	21 (36.8)	3 (2.7)	4 (3.6)	χ^2^ = 12.654 ***p* = 0.013**
No	8 (14.0)	11 (40.7)	8 (32.1)	
Did not know	28 (49.1)	13 (48.1)	15 (53.6)	
	Age ≤ 24 years old (n = 28)	Age 25–30 years old (n = 53)	Age ≥ 31 years old (n = 31)	
Yes	3 (10.7)	11 (20.8)	14 (45.2)	χ^2^ = 11.024 ***p* = 0.026**
No	9 (32.1)	15 (28.3)	4 (12.9)	
Did not know	16 (57.1)	27 (50.9)	13 (41.9)	

**Table 6:** Oral hygiene behavior during pregnancy.

Question	Total (n = 112) n (%)
**How many times a day do you brush your teeth?**	
≤1/d	25 (22.3)
2–3/d	86 (76.8)
≥4/d	1 (0.9)
**How do you take care of your interdental surfaces?**	
Dental floss	51 (45.5)
Interdental brush	10 (8.9)
Irrigator	19 (17.0)
I don’t brush between my teeth	40 (35.7)
Other	1 (0.9)
**Who taught you about proper oral hygiene during pregnancy?**	
Dental hygienist	43 (38.4)
Dentist	44 (39.3)
Obstetrician-gynecologist	4 (3.6)
No one	34 (30.4)
**How often should a pregnant woman see a dental specialist during pregnancy?**	
1 time	37 (33.0)
2 times	11 (9.8)
3 times	1 (0.9)
When the symptoms occur	56 (50.0)
It is not safe during pregnancy	7 (6.3)

## Data Availability

The datasets analyzed during the study are available from the corresponding author upon reasonable request.
